# Infantile Deaths and Poverty

**Published:** 1915-06

**Authors:** 


**Infantile Deaths and Poverty.** Am. Med., April 1915, editorially cites statistics that for incomes of $25 a week and over, the infantile death rate is 84:1000; for those under $10, 256:1000. Of course, the more babies per family, the higher the death rate.

